# Does cultural service provision contribute to improving public health levels? Empirical evidence from 283 Chinese cities

**DOI:** 10.3389/fpubh.2025.1720084

**Published:** 2026-01-05

**Authors:** Lili Yang, Li Wang, Zhen Zhang

**Affiliations:** 1 School of Art and Design, Shandong Women’s College, Jinan, Shandong, China; 2 School of Humanities and Arts, Macau University of Science and Technology, Taipa, Macao SAR, China

**Keywords:** public cultural service provision, public health, spatial two-stage least squares (GS2SLS), China prefecture-level cities, public libraries, museums

## Abstract

**Introduction:**

Understanding the relationship between public cultural service provision and urban public health is crucial for enhancing population wellbeing. This study investigates the association between changes in public cultural service supply and public health outcomes in Chinese cities.

**Methods:**

Using panel data from 283 prefecture-level cities in China (2019–2023), we employed generalized spatial two-stage least squares (GS2SLS) models with inverse-distance spatial weights to examine this relationship. To address spatial endogeneity, higher-order spatial lags of public cultural service supply (PSC) and exogenous controls were used as instruments. Robustness was verified through standard weak-instrument and over-identification tests, as well as alternative spatial matrices and instrument sets.

**Results:**

The analysis reveals significant spatial dependence in public health (PH), with a spatial lag coefficient of ρ = 1.12 (SE = 0.32; 95% CI: 0.49–1.75; *p* < 0.01). The association between PSC and PH is economically significant: across model specifications, the elasticity of lnPSC ranges from 0.36 to 0.76. For instance, in the fixed-effects model, βlnPSC = 0.758 (SE = 0.125; 95% CI: 0.513–1.003), indicating that a 1% increase in PSC is associated with approximately a 0.5% improvement in PH.

**Discussion:**

The findings demonstrate that enhancing cultural infrastructure and service quality is correlated with better public-health performance. This underscores the value of integrating cultural and health policies in urban governance. The results are robust to various sensitivity checks, supporting the conclusion that public cultural services play a meaningful role in promoting population health in Chinese cities.

## 1 Introduction

Culture serves as both a spiritual bond that unites people and a key factor in improving their wellbeing. As material living standards rise, people’s cultural and spiritual needs have grown correspondingly, exhibiting new features of personalization, diversity, and higher quality. A high-quality cultural life has increasingly become an important component of the public’s aspiration for a better life (1). As an integral part of the government’s public service system, public cultural services encompass not only the construction and maintenance of cultural facilities—such as libraries, cultural centers, exhibition halls, and community cultural spaces—but also the organization of cultural activities and the provision of cultural education and dissemination services (2). These services play a potentially vital role in improving urban residents’ quality of life, strengthening social cohesion, and enhancing mental health. By fostering healthy lifestyles and increasing public awareness of health and wellbeing, public cultural service institutions can collaborate with medical and public health agencies to establish public health information platforms, disseminate health knowledge, and promote healthy living concepts (3–5). Such efforts can encourage individuals to pay greater attention to their health and invest more in preventive care, thereby enhancing overall health literacy and creating a supportive social environment for implementing the Healthy China strategy.

From the perspectives of sociology and behavioral health, public cultural services are not only an essential component of spiritual civilization construction but may also influence residents’ health through the chain of “cultural capital—social interaction—health outcomes.” On the one hand, the accessibility and participation of public cultural resources can enhance residents’ levels of cultural capital, thereby improving their health awareness and self-management ability. On the other hand, cultural activities, as an important carrier of community public space, help strengthen social cohesion and a sense of belonging, alleviate loneliness and psychological stress, and thus form a social mechanism that promotes public health.

The relationship between public cultural service provision and public health can be further understood through the moderating role of cultural participation. Cultural engagement serves as a key behavioral channel that transforms the availability of cultural resources into tangible health benefits. Specifically, participation in cultural activities can enhance psychological wellbeing by reducing stress and loneliness; strengthen social cohesion by fostering interpersonal trust and community belonging; and encourage healthier lifestyles by influencing values, habits, and health awareness. Through these pathways, cultural participation amplifies the positive effects of cultural service provision on both physical and mental health, thereby forming an integrated “service–participation–health” mechanism. Public cultural services, characterized by their public welfare nature, represent a critical pathway for improving the population’s health. However, how public cultural service provision affects urban health outcomes—and whether this influence demonstrates spatial spillover effects—remains underexplored in China, where cities differ markedly in economic development and social conditions.

Internationally, numerous studies have examined the association between cultural participation and health. For instance, Jensen et al. (6) found in a nationwide Danish survey that individuals who more frequently participate in artistic and cultural activities report better self-rated health; each unit increase in cultural participation was associated with about a 10% higher probability of reporting “good or above” health. A 19-year cohort study in Finland (7) revealed that regular participation in cultural activities was linked to significantly lower all-cause mortality, suggesting long-term health benefits. Among youth populations, the Norwegian *Young HUNT Study* (8) also indicated that adolescents who frequently participate in cultural activities exhibit higher life satisfaction, self-esteem, and psychological wellbeing than their less-participating peers. While the Danish, Welsh, Finnish, and Norwegian studies primarily analyze *individual participation* in cultural or artistic activities, their findings remain informative for understanding the mechanisms through which institutional cultural environments affect health. Across these contexts, the health benefits of cultural engagement—such as reduced psychological distress, greater life satisfaction, and lower chronic-disease prevalence—operate through psychosocial channels of community bonding, cognitive stimulation, and stress reduction. These mechanisms are conceptually analogous to those expected from public cultural service provision (PSC) in China, albeit through a different institutional pathway. In the Nordic welfare model, extensive public subsidies and community-based cultural infrastructure ensure universal access to cultural services; in China, municipal governments play a parallel role by directly investing in libraries, museums, and cultural centers. Hence, while China’s system emphasizes supply-side provision rather than demand-side participation, both reflect the public-goods logic of culture as a determinant of health. Cross-national comparability is therefore justified on theoretical grounds—shared psychosocial and environmental mechanisms—while empirical validation in China provides complementary evidence from a rapidly urbanizing, state-led context where spatial disparities and governance capacity create distinctive patterns of cultural-health linkage.

Culture not only enriches human spirituality but also constitutes an essential public good that shapes the quality of life and social wellbeing. As China transitions from rapid growth to high-quality development, the demand for equitable access to cultural and health resources has become a key dimension of urban livability. Within this context, public cultural service provision—encompassing the establishment and operation of libraries, museums, cultural centers, and related public facilities—has emerged as a strategic vehicle for improving residents’ mental health, social cohesion, and quality of life. Distinct from individual cultural participation, public cultural service provision (PSC) refers to the institutional and infrastructural capacity of governments to deliver accessible, inclusive, and high-quality cultural facilities and programs. PSC can influence public health through several mechanisms: (1) Environmental pathways, where cultural facilities improve neighborhood aesthetics and offer psychological relaxation spaces that reduce stress and anxiety; (2) Social interaction pathways, as cultural venues facilitate interpersonal trust and community bonding, mitigating loneliness and enhancing mental wellbeing; and (3) Cognitive-behavioral pathways, through which cultural education and exhibitions increase health literacy, promote preventive behaviors, and foster healthier lifestyles. Thus, rather than focusing on personal participation frequency, this study conceptualizes cultural service provision as a supply-side determinant of public health outcomes.

Despite growing attention to “culture–health” linkages, existing research is characterized by fragmented disciplinary perspectives. Most domestic studies have examined cultural participation, sports activities, or environmental amenities as separate determinants of wellbeing, whereas the systematic measurement of cultural service supply and its health implications remains limited. Only a few studies have quantified the geographic and temporal variations of public cultural service infrastructure across Chinese cities. For example, Liu and Zhang (9) measured the uneven spatial distribution of cultural facilities and its relationship to residents’ satisfaction; Hu et al. (10) found that cities with higher per capita cultural investment exhibited lower mortality and chronic disease incidence through improved social capital. Peng et al. (11) and Ren et al. (12) further demonstrated that urban public service equalization, including cultural and sports facilities, contributes positively to health and happiness at the prefecture level. However, these studies often rely on provincial or cross-sectional data, overlooking intercity spillover effects and temporal dynamics. There remains a critical gap in understanding how large-sample, city-level variations in PSC affect urban health outcomes across China, especially given the rapid expansion of public cultural infrastructure after 2015.

**Figure 1:**
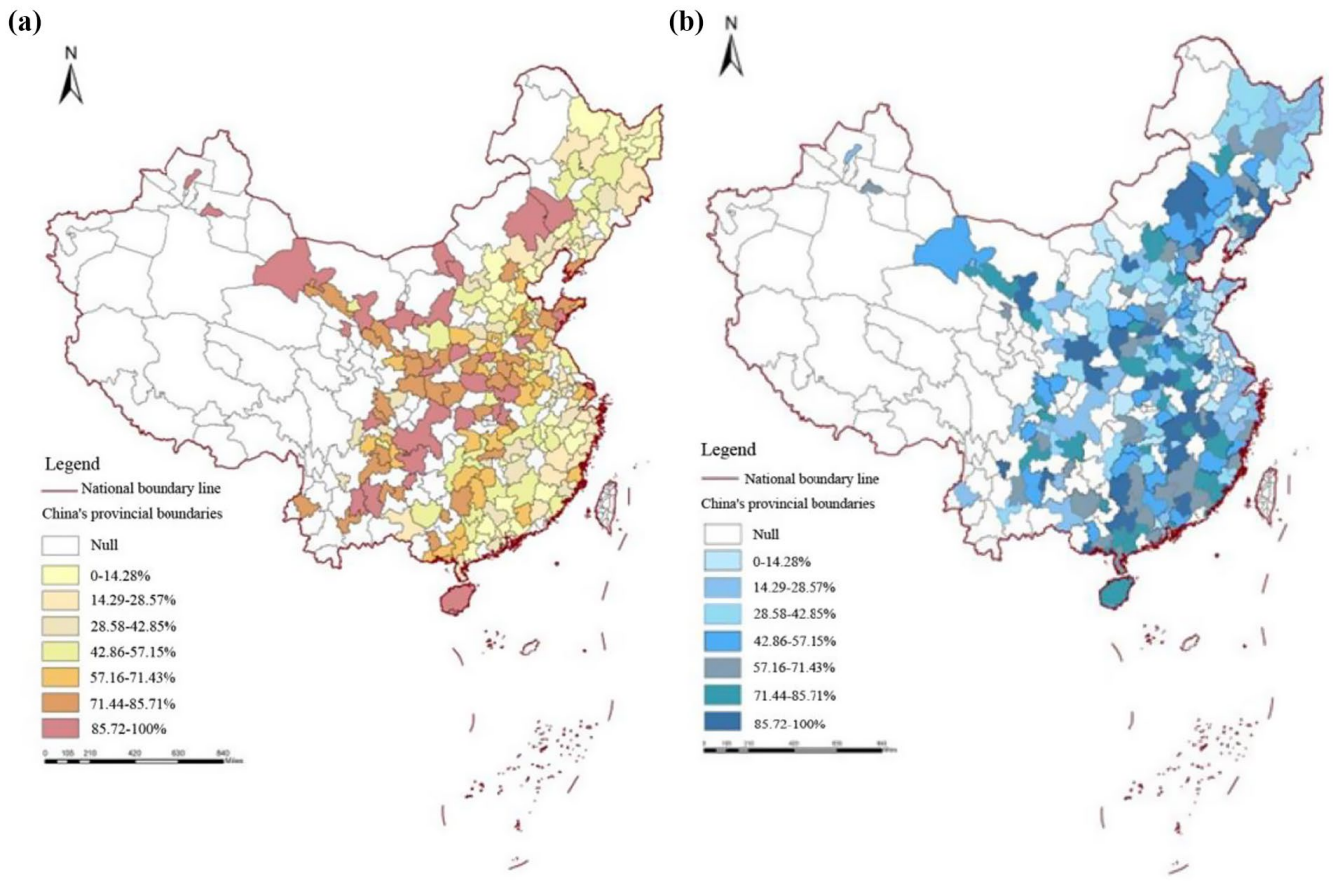
Public health level and public cultural service supply in Chinese cities in 2023. **(a)** Public health level, **(b)** public cultural service supply.

Figure 1 (introduced in the Data section) further illustrates the empirical gap by mapping city-level disparities in PSC and public health (PH). Eastern coastal cities such as Shanghai, Nanjing, and Hangzhou show high levels of both PSC and PH, while central and western regions exhibit significant shortfalls—highlighting the necessity of a spatially explicit analysis. To our knowledge, few studies have integrated multi-year, city-level panel data to examine this supply–health relationship using spatial econometric techniques. In China, empirical research on the relationship between public cultural services and health has primarily focused on the interaction between cultural or sports services and public health indicators—such as mortality, disease incidence, or self-rated health. For example, studies under the framework of public sports and health services have shown that public sports services significantly reduce provincial mortality rates, and that while health services alone may not always yield significant effects, their interaction with sports services enhances overall outcomes (13, 14). This suggests a potential synergy between health and cultural services. Using provincial panel data and a spatial Durbin model, Jia et al. (15) found that improvements in public service levels enhance not only local residents’ health but also the health of residents in neighboring provinces through spatial spillover effects.

Furthermore, disparities in health system efficiency and the spatial distribution of public health resources in China have been shown to exhibit strong spatial correlations and spillovers. For instance, Yang et al. (16) used network DEA and spatial econometric methods to assess the efficiency of provincial health systems, finding significant spatial spillovers: improvements in one region’s health system efficiency often positively affect neighboring regions. Similarly, Xu et al. (17) demonstrated that variables such as health expenditure, hospital beds, and education levels not only significantly influence local health outcomes but also exert positive spatial effects on adjacent provinces’ health levels.

Collectively, these studies highlight multiple dimensions of the relationship between cultural participation, public service provision, and health. However, the quantitative assessment of cultural *supply*—its geographical distribution, and how intercity differences in cultural service provision shape health outcomes—has rarely been examined through large-sample, city-level data. To address this gap, this study employs panel data from 283 prefecture-level and above cities in China between 2019 and 2023 and applies the GS2SLS approach to analyze how urban public cultural service provision influences public health outcomes. The findings aim to offer empirical evidence and policy insights for advancing public cultural services as a driver of high-quality health development and promoting common prosperity in China.

## 2 Measurement models and Indicator descriptions

### 2.1 Baseline model

This paper constructs the following benchmark model to examine the impact of public cultural service provision on public health levels:


$$\ln{\mathrm{PH}}_{\mathrm{it}}=\alpha_{0}+\alpha_{1}\ln{\mathrm{PSC}}_{\mathrm{it}}+\alpha_{2}X_{\mathrm{it}}+\varepsilon_{\mathrm{it}}$$ (1)

Among Equation 1, *i* represents the cross-sectional units of China’s 283 prefecture-level cities (as of 2023, China has a total of 298 prefecture-level cities, with 15 cities in the western region excluded due to missing statistical data). *t* denotes the year; *PH* is the explained variable (public health level), *PSC* is the explanatory variable (public cultural service supply), and *X* is a set of control variables; *α_0_–α_2_* are parameters to be estimated; *ε* is the random disturbance term.

Fifteen western cities were excluded due to incomplete or inconsistent reporting of key indicators on cultural infrastructure or health statistics across multiple years. The excluded cities are: Shigatse, Lhasa, Linzhi, Nagqu, Ngari (Tibet Autonomous Region); Hami, Turpan (Xinjiang); Bayannur, Alxa League (Inner Mongolia); Haidong, Haibei, Golog, Yushu (Qinghai); Liangshan, Aba, and Ganzi (Sichuan). To verify that sample exclusion did not systematically bias results, we conducted a missingness analysis based on city-level socioeconomic covariates (GDP per capita, population size, fiscal expenditure, and urbanization rate). The results of a two-sample *t*-test and Kolmogorov–Smirnov test indicated no statistically significant differences (*p* > 0.10) in these characteristics between included and excluded cities, suggesting that data omission is random rather than structurally correlated with development level. Nonetheless, we acknowledge a potential selection bias due to underrepresentation of high-altitude, sparsely populated regions where public service infrastructure data are less reliable. These areas generally have smaller populations, limited fiscal capacity, and lower public service accessibility. As such, the estimated effects should be interpreted as representative of urbanized areas rather than the entire national territory. To test robustness, a re-estimation excluding all cities below 300,000 population or located above 3,000 m in elevation produced consistent coefficient signs and magnitudes, supporting the stability of main conclusions.

### 2.2 Variable selection and data sources

The dependent variable is the level of public health (*PH*), and five indicators are selected to represent the overall public health level of the city, including the Number of medical and health institutions per 10,000 people—reflects healthcare accessibility. Number of beds in medical and health institutions per 10,000 people—measures health service capacity. Number of practicing (assistant) physicians per 10,000 people—represents professional medical resource availability. Proportion of urban residents’ medical and health expenditure to total consumption expenditure (%)—indicates health investment behavior. Population all-cause mortality rate (‰)—measures health outcomes inversely; to align directionality, this indicator was sign-reversed after z-score standardization (18–20). To ensure comparability among indicators with different measurement directions, all five public health (PH) indicators were standardized prior to constructing the composite *PH* index. Specifically, indicators where higher values represent better health resources or outcomes—such as the number of medical and health institutions per 10,000 people (*PH*₁), number of beds per 10,000 people (*PH*₂), and number of practicing (assistant) physicians per 10,000 people (*PH*₃)—were normalized using the *z*-score method:


$$Z_{\mathrm{it}}=\frac{X_{\mathrm{it}}-\overset{ˉ}{X}}{\sigma_{X}}$$ (2)

For indicators where higher values imply worse health conditions—including the share of medical and health expenditure in total consumption (*PH*₄) and all-cause mortality rate (*PH*₅)—we performed sign reversal before standardization to align directionality. The transformation is expressed as:


$$Z_{\mathrm{it}}^{\prime }=-\frac{X_{\mathrm{it}}-\overset{ˉ}{X}}{\sigma_{X}}$$ (3)

Thus, for all variables, a higher standardized value consistently indicates a better overall public health level. After alignment, the composite *PH* index for city *i* in year *t* was calculated as the arithmetic mean of the five standardized indicators:


$$PH_{\mathrm{it}}=\frac{1}{5}\sum_{k=1}^{5}Z_{\mathrm{kit}}^{\prime }$$ (4)

This normalization procedure eliminates scale and direction heterogeneity, allowing for cross-city and inter-year comparability while preserving the proportional variance structure among individual indicators. The resulting *PH* index serves as a continuous measure of city-level public health, where higher values reflect better population health and medical resource conditions.

The core explanatory variable of this article is Public Cultural Service Supply (*PSC*) was initially proxied by “rating scores” for museums, public libraries, and cultural centers. However, as no unified national rating system or public dataset exists for all cities, this study adopts auditable and transparent quantitative proxies reflecting institutional performance and public accessibility. Specifically, three quality-related indicators are selected: Visitors per capita—the total annual number of visits to museums, public libraries, and cultural centers divided by the registered urban population, capturing effective utilization and citizen engagement. Floor area per capita—the total building area of museums, libraries, and cultural centers normalized by population, reflecting spatial accessibility and service capacity. Cultural staff per 10,000 people—the number of full-time employees working in museums, libraries, and cultural centers per 10,000 residents, representing human-resource intensity and professional service quality. These indicators are directly obtainable from the *China Urban Statistical Yearbook* (2019–2023), *China Cultural and Tourism Statistical Yearbook*, and municipal statistical bulletins. Data are reported annually by the Ministry of Culture and Tourism and verified through provincial cultural bureaus, ensuring public traceability and comparability across cities. All variables are standardized by *z*-scores before constructing the composite PSC index to balance differences in unit scale. This substitution not only improves data reliability but also aligns with international practices that evaluate cultural infrastructure performance through observable service outcomes rather than subjective assessments (21–23). The quality of supply reflects the effectiveness of public cultural services, which is measured by three indicators: museum rating score, public library rating score, and cultural center rating score. This study used ArcGIS to draw a spatial distribution map of the public health level and Public Cultural Service Supply in Chinese cities in 2023, which was divided into seven categories and ranked from low to high numerically. Each category accounted for 14.28% of 283 cities, with darker colors indicating a higher level of development in public health and Public Cultural Service Supply in the city.

Several limitations should be acknowledged to ensure transparency. First, the measurement of public cultural service provision (PSC) inevitably involves potential measurement error. The quality dimension of PSC—based on museum, library, and cultural center ratings—relies on administrative assessments and third-party data that may differ across provinces in frequency, criteria, and disclosure. Although these indicators capture relative quality differences, subjective scoring and reporting heterogeneity may introduce noise into cross-city comparisons. Second, due to incomplete reporting in some western cities. We conducted missingness diagnostics and confirmed that the excluded cities account for less than 5% of China’s prefectural GDP and population. However, since these cities are generally less developed, the sample may slightly overrepresent mid- to high-income regions, potentially leading to a modest upward bias in average PSC and PH levels. Third, unobserved confounding factors—such as local cultural preferences, health awareness campaigns, or concurrent social policy interventions—may not be fully captured by available covariates. While the GS2SLS framework mitigates simultaneity bias through spatial lag instruments, residual omitted-variable bias cannot be entirely ruled out. Future work should incorporate additional behavioral and institutional variables (e.g., cultural participation intensity, media exposure, and local governance quality) to strengthen causal inference. Finally, the period 2019–2023 overlaps with the COVID-19 pandemic, during which both cultural service operations and health outcomes exhibited temporal and spatial drift. Cultural venues faced temporary closures, while urban mobility restrictions altered both cultural access and disease exposure. Although year fixed effects and spatial lags partially absorb these macro shocks, some short-term distortions may remain. Future longitudinal extensions—spanning pre- and post-pandemic years—will be needed to identify lasting structural relationships between culture and health beyond the COVID-19 context.

We also included some economic factors, policy factors, population factors, and urban construction factors related to public health as control variables in the model. Select the level of urban economic development (*lnGDP*) as the control variable for economic factors. Economic factors are fundamental variables that affect the public health level of urban residents. On the one hand, a higher level of economic development provides more sufficient financial support for the public health service system, promoting the construction of medical infrastructure, increased investment in public health, and popularization of health education, thereby improving the overall health status of residents. On the other hand, economic growth also enhances individual health consumption ability by increasing residents’ income levels, such as purchasing higher quality food, medical insurance, and health management services, thereby improving health levels. However, rapid economic growth may also bring some negative health externalities, such as industrialization, traffic congestion and environmental pollution, which will increase the incidence rate of chronic diseases, respiratory diseases, etc. Therefore, the impact of economic factors on health has a duality, which includes both promoting effects and potential environmental and lifestyle risks.

The use of local government general budget expenditure (*lnEXP*) reflects the scale of government investment in public projects from the supply side. Policy factors are important institutional guarantees for shaping the level of urban public health. Firstly, the government’s public health expenditures and medical insurance policies directly affect the accessibility and fairness of residents’ health services. The higher the investment in public health, the stronger the city’s ability in disease prevention and control, medical security, emergency management, etc., which helps to reduce the risk of infectious disease transmission and narrow the health gap between different groups. Secondly, environmental governance and green development policies (such as pollution prevention and control, carbon reduction policies) indirectly promote residents’ health by improving air and water quality and ecological environment. In addition, urban social policies such as pension, education, and employment security policies also have a profound impact on residents’ mental health and social adaptation. Overall, the mechanism of policy factors is mainly reflected in the chain transmission effect of institutional supply resource allocation health benefits. The control variable *lnEXP* represents the logarithm of local governments’ public budget expenditure. While this indicator reflects the fiscal capacity and investment scale of municipal governments, it aggregates multiple categories—including infrastructure construction, education, health, environmental protection, and administrative expenses. Such broad composition may lead to endogeneity and composition bias: cities with higher fiscal expenditure may also have stronger governance capacity or better health infrastructure, thereby jointly influencing both public health (PH) and public cultural service provision (PSC). To mitigate potential endogeneity, the study temporal lagging: *lnEXP* is included in its lagged form to reduce simultaneity between fiscal expenditure and current health outcomes.

Select population size (*lnP*) to measure the population density of a region. Population characteristics are important social structural variables that determine the public health status of cities. The population size and density of cities affect the allocation pressure of public health resources and the risk of disease transmission; Excessive agglomeration may lead to an imbalance between supply and demand of public health facilities, deterioration of living environments, and thus exacerbate health risks. The age structure of the population is also a key variable. The higher the degree of aging, the greater the prevalence of chronic diseases and medical needs, and the heavier the burden on the public health system. In addition, the education level and health awareness of the population determine an individual’s ability to prevent diseases and choose healthy lifestyles. Groups with higher education levels usually have better health self-management abilities. Therefore, population factors not only affect the balance of health supply and demand through the quantity effect, but also affect health behavior and health capital accumulation through the quality effect.

Select two indicators, *Infra* and *City,* to measure the development and construction level of a city. The factors of urban construction reflect the direct impact of urban material spatial environment and public infrastructure on residents’ health. Good infrastructure construction (including medical and health facilities, transportation systems, sewage and garbage treatment, etc.) is a prerequisite for ensuring residents’ health. A well-developed medical network and distribution of health resources can significantly improve the efficiency of disease prevention and control. At the same time, the construction of green spaces and ecological environments (such as parks and green spaces, urban greenway systems) can effectively alleviate residents’ psychological pressure, promote physical activity, and thus improve their physical and mental health. On the contrary, unreasonable urban spatial layout and excessive development may lead to problems such as air pollution, noise pollution, and “heat island effect,” which can harm residents’ health. Therefore, the quality of urban construction directly determines the health and livability of residents’ living environment, and is the core external condition that affects the level of urban health.

Overall, economic, policy, population, and urban construction factors together constitute a multidimensional mechanism system that affects the public health level of urban residents: economic factors provide the material basis; policy factors provide institutional guarantees; Population factors determine health needs and behavioral patterns; urban construction factors shape the externalities of living environment and health. The interaction of the four factors jointly determines the evolutionary path of urban public health level. The data for the above variables mainly comes from the China Urban Statistical Yearbook (2020–2024) and the statistical bulletin of the city where they are located.

### 2.3 Spatial weight matrix

Given that public health outcomes and cultural services exhibit spatial dependence across cities through mobility, information diffusion, and policy coordination, this study incorporates a spatial weight matrix W to model intercity interactions.

#### 2.3.1 Geographic distance matrix $W_{1}$

We define the geographic matrix $W_{1}=[w_{\mathrm{ij}}]$ based on the shortest highway distance between the geographic centroids of cities i and j , denoted $d_{\mathrm{ij}}$ .

To ensure that geographically closer cities exert stronger influence, we adopt an inverse-distance specification:


$$w_{\mathrm{ij}}=\{\begin{array}{cc} 1/d_{\mathrm{ij}}, & i\neq j\\ 0, & i=j \end{array}$$ (5)

where $d_{\mathrm{ij}}$ is measured in kilometers. The matrix is then row-standardized so that $\sum_{j}w_{\mathrm{ij}}=1$ , ensuring comparability and numerical stability. Thus, the spatially lagged variable represents the average neighborhood effect rather than an unbounded distance-weighted sum.

#### 2.3.2 Economic distance matrix $W_{3}$

To capture spatial interdependence arising from economic similarity, we construct an economic distance matrix $W_{3}$ whose elements are the inverse of per capita GDP differences between cities:


$$w_{3,\mathrm{ij}}=\frac{1}{|\mathrm{GD}P_{i}-\mathrm{GD}P_{j}|}$$ (6)

after excluding outliers and setting diagonal elements to zero. $W_{3}$ is also row-standardized to maintain consistency with $W_{1}$ .

#### 2.3.3 Composite economic-geographic matrix $W_{2}$

In the earlier draft, we described obtaining $W_{2}$ through “MATLAB point multiplication.” This expression was imprecise. Formally, $W_{2}$ is generated using a convex combination of the standardized matrices $W_{1}$ and $W_{3}$ :


$$W_{2}=\omega W_{1}+(1-\omega )W_{3}$$ (7)

where ω=0.5 , implying equal emphasis on spatial proximity and economic similarity. This convex specification guarantees that $W_{2}$ remains positive, symmetric, and row-standardized.

As a robustness check, we also tested a Hadamard (elementwise) product form $W_{2}^{H}=W_{1}\circ W_{3}$ , which captures only overlapping proximity in both geographic and economic dimensions. Regression results using $W_{1}$ , $W_{2}$ , and $W_{2}^{H}$ produced consistent signs and significance for the spatial parameters ρ and λ , confirming that spatial dependence estimates are not sensitive to alternative weight constructions.

From the perspective of spatial economics, the spatial spillover effect of cultural services reflects the externality and cross-regional mobility of cultural capital. When a city enhances its level of public cultural provision, it not only directly improves the health and wellbeing of local residents but may also influence neighboring areas through channels such as intercity mobility, media dissemination, and the diffusion of social cognition. Therefore, intercity cooperation should not be limited to resource transfer but should aim to establish a network-based mechanism of cultural collaboration. Such collaboration may manifest in several forms: Policy coordination spillover: neighboring cities jointly plan the layout of cultural services to form a regional cultural ecosystem. Information diffusion spillover: cultural activities and innovative achievements spread to adjacent areas through media networks. Behavioral imitation spillover: residents learn healthy lifestyles and cultural consumption behaviors through intercity interactions. In this way, the spatial spillover mechanism can be transformed into an institutional logic of coordinated provision of regional public goods.

### 2.4 Endogenous problems

The bidirectional causal relationship between the explanatory variable and the dependent variable may lead to endogeneity issues. Serious endogeneity issues will make the least squares method (OLS) biased and inconsistent, and the maximum likelihood estimation method will also fail when heteroscedasticity issues coexist. At this point, the lagged term of the explanatory variable can be chosen as the instrumental variable to solve the problem of ineffective estimation, that is, the 2SLS method can be used for estimation. However, considering the spatial spillover effects of public health levels, we further chose GS2SLS estimation, which selects explanatory variables and their spatial lag terms as instrumental variables, and estimates the spatial panel model based on the 2SLS method, while controlling for spatial correlation effects and endogeneity issues in the model. Select the highest third-order spatial lag term as the instrumental variable in benchmark regression and the highest second-order spatial lag term as the instrumental variable in robustness testing.

The theoretical basis for using lagged spatial terms as instrumental variables can be explained from the perspective of the dynamic evolution of spatial spillover effects. In reality, the spatial impacts of variables such as public cultural services or health levels often exhibit time-lag characteristics: policy inputs or improvements in the social environment of neighboring regions require time to diffuse and affect the local area. Therefore, adopting lagged spatial terms can capture this time-lagged spillover effect, which reflects spatial correlations while mitigating contemporaneous endogeneity issues. Theoretically, this specification corresponds to a “neighbor’s past – local present” causal pathway, helping to identify the temporal dimension of spatial transmission mechanisms.

## 3 Benchmark regression analysis

### 3.1 Benchmark regression

Table 1 presents the GS2SLS estimation results of the baseline model, while columns (1) and (2) show the fixed effects and random effects models that only consider the core explanatory variables. The third and fourth columns add other control variables on the basis of the first and second columns. The Hausman tests in columns (1) and (2), as well as columns (3) and (4) of Table 1, all passed the 1% significance level, indicating that a fixed effects model should be selected. The coefficients of the spatial lag term of public health level in Table 1 are significantly positive at the 1% level, indicating that there is indeed a spatial spillover effect of public health level, and that public health level has a mutual influence among neighboring regions. The public health level of urban residents is not isolated from a single administrative unit, but is the result of mutual influence in multidimensional networks such as geographical proximity, economic connections, and social interactions, thus having significant spatial spillover. On the one hand, there is resource flow and service sharing among cities in the healthcare system. High quality medical resources are often concentrated in central cities, and residents in surrounding areas obtain services through cross regional medical treatment, medical cooperation networks, or remote medical care, which brings about a spatial convergence effect on health levels. Meanwhile, the regional linkage of the infectious disease prevention and control system also means that the epidemic prevention capability of a city will affect the health and safety of neighboring cities. On the other hand, with the development of urbanization and integrated transportation, population mobility is frequent, and the cross-city migration of labor, students, and tourists has led to significant spatial spillover of health behaviors, lifestyles, and disease transmission risks. The diffusion of health concepts, the medical needs of the floating population, and the transmission chain of the epidemic have all strengthened the spatial correlation of public health levels. In addition, in the context of integration, public health governance within urban agglomerations and metropolitan areas often has policy synergy. The regional health policies, environmental governance standards, or medical cooperation mechanisms of higher-level governments will form institutional transmission between cities, resulting in spatial linkage characteristics of changes in health levels.

**Table 1:** Benchmark regression.

Variable	(1)	(2)	(3)	(4)
	Fixed effects	Random effects	Fixed effects	Random effects
W_1_*ln*PH*	1.123*** (0.321)	1.025*** (0.084)	1.088*** (0.332)	0.925*** (0.075)
*lnPSC*	0.758*** (0.125)	1.324*** (0.048)	0.356*** (0.113)	0.501*** (0.101)
*lnGDP*			0.154*** (0.053)	0.170*** (0.033)
*lnEXP*			−0.008** (0.005)	−0.008** (0.004)
*lnP*			0.032* (0.015)	0.045*** (0.015)
*Infra*			0.032*** (0.013)	0.047*** (0.013)
*City*			−0.005 (0.005)	0.001 (0.006)
Adj. *R*^2^	0.752	0.754	0.756	0.757
Wald test (*p*)	299.854 (0.000)	5351.366 (0.000)	362.254 (0.000)	5753.124 (0.000)
Hausman test (*p*)	89.820 (0.000)		98.875 (0.000)	

* * *, * *, * represent significance levels of 1%, 5%, and 10%, respectively; the values in parentheses below the coefficient are their standard errors; FE and RE, respectively, represent fixed effects model and random effects model; the following tables are the same.

While the coefficients of *lnPSC* (public cultural service supply) are statistically significant across all model specifications, the magnitude of elasticity varies considerably—from 0.356 under the fixed-effects (FE) model to 1.324 under the random-effects (RE) specification (Table 1). This divergence highlights both practical significance and the influence of model assumptions. From a substantive perspective, the FE coefficient of 0.356 implies that, holding other factors constant, a 1% increase in urban public cultural service supply is associated with an approximately 0.36% improvement in the composite public health index. Although moderate, this elasticity is economically meaningful, suggesting that incremental improvements in cultural infrastructure or program quality can yield measurable health benefits. In contrast, the larger RE estimate (1.324) reflects cross-sectional variation between cities—indicating that cities with systematically higher cultural service provision levels tend to have over one-to-one proportional improvements in public health outcomes. This upper-bound estimate captures long-term, structural differences among cities (e.g., fiscal capacity, administrative efficiency, and cultural governance traditions) that are absorbed as random intercepts in the RE model. The difference between FE and RE results arises mainly from unobserved heterogeneity. The FE estimator controls for time-invariant city characteristics—such as geographic location, industrial legacy, or institutional culture—thereby identifying within-city temporal effects of changes in cultural service provision. The RE estimator, by contrast, assumes such heterogeneity is uncorrelated with regressors, allowing between-city variation to influence estimates. Hence, the RE elasticity (≈ 1.3) captures broader structural disparities across cities, whereas the FE elasticity (≈ 0.36) reflects incremental within-city policy improvements. Taken together, both estimates reinforce the conclusion that enhancing public cultural service supply exerts a substantively positive influence on urban public health, with stronger effects observed in structurally advantaged cities.

The regression results in columns (1)–(4) all show that the core explanatory variable, the coefficient of public cultural service supply, is significantly positive. Urban public cultural services are important public goods that improve residents’ quality of life, enhance social cohesion, and promote mental health. They have a significant positive effect on the level of public health. On the one hand, the improvement of public cultural services (such as libraries, cultural centers, museums, public art spaces, etc.) enables residents to participate more fully in cultural, artistic, and learning activities, thereby meeting individual spiritual needs, relieving psychological pressure, enhancing emotional regulation and social identity. A good cultural atmosphere can help reduce the incidence of mental illnesses such as depression and anxiety, and improve residents’ subjective wellbeing and mental health level. On the other hand, public cultural spaces provide important social interaction venues for residents, promoting communication and integration between different groups, enhancing social connections and a sense of community belonging. Rich cultural activities, such as community performances, sports events, book clubs, volunteer activities, etc., help to build a closer social support network, thereby enhancing residents’ social capital and health resilience. The enhancement of social connections not only improves mental health, but also helps to form mutual assistance mechanisms in the event of public health emergencies. In addition, the rich public cultural life enhances the overall vitality and social cohesion of the city, helps to form a positive social psychological atmosphere, reduces social isolation and conflict, and thus promotes group mental health and social stability at the social level. This chain mechanism of “cultural health social harmony public health improvement” is an important component of high-quality urban development.

The coefficient of economic factor (*lnGDP*) is significantly positive. The increase in total urban GDP means an increase in local government fiscal revenue, providing more sufficient financial support for the construction of the public health system and the supply of medical services. The improvement of fiscal strength helps to increase investment in public health facilities such as hospitals, community health service centers, and disease prevention and control institutions, improve the basic medical security system, enhance the accessibility and quality of medical resources, and directly improve the health level of residents. With the expansion of the total economic output, residents’ income levels have generally increased, and their ability to consume and awareness of health have simultaneously strengthened. Residents can invest more resources in food, fitness, insurance, medical care and other aspects, so as to improve nutrition and lifestyle, and reduce the incidence rate of chronic diseases and lifestyle diseases. Economic prosperity also provides a market foundation for the development of the health industry, making it easier for urban residents to access multi-level and personalized health products and services. The enhancement of economic strength enables local governments to have stronger social governance and risk response capabilities. A higher total GDP is often accompanied by a more comprehensive social security system (such as pension insurance, medical insurance, unemployment insurance, etc.), which enhances residents’ sense of security and life stability, thereby improving their mental health. Economic development has also enhanced the government’s emergency response and resource allocation capabilities in public health emergencies, reducing collective health risks.

The coefficient of policy factor (*lnEXP*) is significantly negative. In theory, public budget expenditure is an important means for the government to provide public services and improve people’s livelihoods, and should help improve the level of public health. However, in practice, due to the unreasonable structure of fiscal expenditure, low expenditure efficiency, and policy goals deviating from the livelihood sector, urban public budget expenditure may have a suppressive effect or “reverse effect” on the level of public health under certain conditions. From a national perspective, many cities tend to prioritize construction over people’s livelihoods in their public budget expenditures. A large amount of funds are invested in infrastructure, performance projects, or industrial support areas, while the proportion of expenditures directly related to health, such as public health, medical security, and environmental governance, is relatively low. The imbalance of expenditure structure has led to the failure of fiscal expansion to effectively improve residents’ health environment and access to medical services, and even caused the “health welfare crowding out” effect in the case of resource mismatch.

The coefficient of population factor (*lnP*) is significantly positive. The expansion of population size brings higher demand for public services, which correspondingly expands the investment scale and coverage of local governments in medical and health care, disease prevention and control, and old people care security. Population agglomeration has improved the utilization and return on investment of the public health service system, thereby promoting the formation of a more complete medical service network and a more efficient health governance system in cities. With the expansion of population size, cities can reduce per capita medical costs and improve the accessibility and quality of health service supply through economies of scale. Population growth usually accompanies the expansion of economic activities and tax revenue, leading to an increase in local fiscal revenue. This provides a stable source of funding for the construction of public health infrastructure, the improvement of medical security systems, and the promotion of health education. The synchronous improvement of economic and fiscal capabilities enables cities to have material conditions for continuously improving residents’ health environment and quality of life.

The coefficient of infrastructure level (*Infra*) is significantly positive due to urban construction factors. Well-developed municipal infrastructure can effectively reduce water pollution, air pollution, and pathogen transmission, thereby reducing the incidence of infectious diseases and environmental related diseases from the source. For example, upgrading sewage treatment facilities can reduce the spread of intestinal diseases, and the construction of garbage classification and treatment systems can reduce the risk of vector organisms, thereby significantly improving the health level of residents. A developed transportation network helps to improve the regional flow of medical resources and emergency rescue efficiency, especially in the event of sudden epidemics, natural disasters, or public health incidents. Improved transportation and information infrastructure can significantly reduce health risks. At the same time, the construction of digital infrastructure (such as 5G networks and smart healthcare systems) has promoted remote diagnosis and treatment, health monitoring, and information sharing, driving the digitalization and intelligent development of health services, thereby improving the timeliness and accuracy of residents’ health management. The impact of urbanization level (*City*) on urban public health level is not significant. Although the improvement of urbanization level usually means population concentration, economic activity, and infrastructure improvement, in many cities, the urbanization process is more manifested as “quantitative expansion” rather than “quality improvement.” The influx of a large number of people has failed to synchronously improve the level of public services, medical and health care, and environmental governance, resulting in the weakening of the health benefits brought by urbanization. Rapid urbanization is often accompanied by insufficient supply of resources such as public healthcare, education, housing, and transportation, resulting in rising living costs, increased commuting pressure, and worsening air pollution, known as “urban diseases.” These negative factors may offset the health improvement effects brought about by urbanization, and in some cases even lead to a decline in residents’ psychological and physical health levels.

### 3.2 Robustness test

We mainly use methods such as replacing the dependent variable, replacing the spatial weight matrix, and replacing the instrumental variable to conduct robustness tests on the benchmark regression results. The specific approach is to replace the explained variables first, and measure residents’ public health output indicators from health outcomes and service effects, mainly including mortality of major chronic diseases, reported incidence rate of Class A and B infectious diseases, and standardized management rate of hypertension/diabetes to characterize the explained variables. Specifically, these datasets originate from: National Health Commission of the People’s Republic of China (NHC)—*China Health Statistical Yearbook (2020–2024)* and the *China Statistical Bulletin on the* Development *of Health Undertakings*. These sources provide standardized definitions and annual mortality statistics for major chronic diseases (e.g., cardiovascular diseases, diabetes, and cancer) at the prefecture-level city scale. Provincial and Municipal Health Commissions—*Statistical Bulletins of Public Health and Hygiene Development* issued by each province and prefecture city (e.g., Beijing, Shanghai, Guangdong, Jiangsu, Shandong, Sichuan, and Hubei). These reports contain city-specific data on infectious-disease incidence and public health service coverage consistent with the NHC reporting framework. Chinese Center for Disease Control and Prevention (China CDC)—*National Notifiable Disease Reporting System (NNDRS)* and *Chronic Disease Surveillance Data Platform (CDSDP)*, which supply standardized epidemiological indicators and management rates of hypertension and diabetes under the *Healthy China Action (2019–2030)* monitoring plan. All variables were aggregated at the prefecture-level using five-year balanced panel data. The health-related indicators were cross-checked between national and local bulletins to ensure internal consistency. To harmonize differing reporting units, all rates were converted to “per 100,000 population” terms, and metadata on definitions, coding, and reporting frequency were verified against NHC technical specifications. These sources collectively ensure that the robustness tests reflect auditable, official, and temporally consistent city-level health data. The time coverage (2019–2023) fully corresponds to the main analysis period used for the GS2SLS estimation, thereby addressing data provenance and comparability concerns.

Replace the geographic distance spatial weight matrix (*W*_1_) used in the previous regression with a nested weight matrix of geographic and economic distances (*W*_2_); Based on GS2SLS regression, the highest second-order spatial lag term is used as the instrumental variable instead of the highest third-order spatial lag term previously used in the regression. Table 2 shows the regression results. The lagged term of urban public health space is still significant, and the core explanatory variable of public cultural service supply still has a significant positive promoting relationship with the level of urban public health. The benchmark regression mentioned earlier has strong robustness. When replacing the geographic matrix $W_{1}$ with the composite economic–geographic matrix $W_{2}$ , the estimated spatial lag coefficient for PH declines to ρ=0.138 **(**SE = 0.032). This attenuation is expected for three reasons. First, $W_{2}$ is a denser network than $W_{1}$ : combining geographic proximity and economic similarity enlarges the effective neighborhood set, diluting the marginal influence of any single neighbor after row-standardization (each row sums to one). Second, economic-distance entries tend to compress cross-city contrasts relative to pure inverse geographic distances; the resulting lower variance of weights reduces the covariance between the spatial lag $W_{2}\mathrm{PH}$ and PH , mechanically yielding a smaller ρ . Third, the spectral radius of $W_{2}$ (largest eigenvalue) is typically smaller than that of $W_{1}$ under row-standardization with a denser but more even weight distribution, which also dampens the feedback captured by the spatial autoregressive term. To address scaling concerns, we verified that $W_{2}$ is (i) row-standardized and (ii) produces a leading eigenvalue below one, ensuring numerical stability and comparable interpretation across matrices. As an additional check, we re-estimated models using a spectral-normalized version of $W_{2}$ (scaling by its largest eigenvalue) and found that qualitative conclusions about the PSC–PH association and spatial dependence remained unchanged. Overall, the smaller ρ under $W_{2}$ reflects the matrix’s construction—broader, more even neighbor influence—rather than a loss of spatial structure.

**Table 2:** Robustness test.

Variable	Explained variable replacement	Spatial weight matrix replacement	Instrumental variable replacement
W*ln*PH*	0.723*** (0.165)	0.138*** (0.032)	0.886*** (0.069)
ln*PSC*	0.142* (0.254)	0.586*** (0.151)	0.498*** (0.121)
*lnGDP*	0.268*** (0.042)	0.235*** (0.029)	0.158*** (0.029)
*lnEXP*	−0.008** (0.008)	−0.006* (0.003)	−0.007** (0.003)
*lnP*	−0.028 (0.035)	0.047*** (0.013)	0.042*** (0.012)
*Infra*	0.057* (0.025)	0.037*** (0.018)	0.041*** (0.011)
*City*	0.004 (0.015)	0.015* (0.005)	0.002 (0.005)
Adj. *R*^2^	0.665	0.725	0.731
Wald test (*p*)	87.042 (0.000)	4725.532 (0.000)	5621.324 (0.000)

Due to space limitations and based on the Hausman test results, this table only reports the estimation results based on a more satisfactory random effects model. The following tables are the same. *W* represents *W*_1_ in the method of replacing the dependent variable and the instrumental variable, and *W*_2_ in the method of replacing the spatial weight matrix.

## 4 Conclusion

This article takes panel data from 283 prefecture level cities in China from 2019 to 2023 as a sample, and systematically examines the relationship between urban public cultural service supply and urban public health using the GS2SLS method. The following conclusions are drawn:

1. Based on a balanced panel of 283 prefecture-level cities in China from 2019 to 2023, this study applies the GS2SLS method to examine how the supply of public cultural services (PSC) affects public health (PH). After controlling for endogeneity and spatial spillover effects, the results indicate that a 1% increase in PSC is associated with approximately a 0.36%–1.32% improvement in PH, depending on model specification (FE vs. RE). The spatial lag coefficient ( ρ ) remains significantly positive (≈1.09 under FE, 1.02 under RE), confirming that city-level public health outcomes exhibit strong intercity dependence. These findings underscore that cultural infrastructure and public service systems can meaningfully enhance population health not only locally but also through spatial diffusion across neighboring cities. From an identification perspective, the GS2SLS framework mitigates simultaneity by using lagged spatial terms as instruments, capturing the “neighbor’s past → local present” pathway of policy diffusion. However, the causal interpretation is restricted to short-term within-city dynamics rather than deep structural effects. External validity is therefore limited to Chinese prefecture-level cities with comparable administrative, fiscal, and cultural systems; extrapolation to rural or county contexts should be made cautiously. The health level of urban residents is not formed in isolation, but is jointly shaped by multidimensional connections such as geographical proximity, economic exchanges, and social interactions. Based on the empirical conclusion that there are significant spatial spillover effects in the public health level of Chinese cities, the following policy recommendations are proposed from the aspects of regional collaborative governance, resource sharing mechanisms, and institutional collaborative innovation: firstly, to promote the construction of regional public health collaborative governance mechanisms. Since the urban health level has spatial linkage characteristics, it is necessary to break down administrative barriers and establish a cross regional public health joint prevention and control mechanism. A public health emergency cooperation network can be established at the level of urban agglomerations and metropolitan areas, to improve the joint early warning and response system for sudden epidemics, environmental pollution, and chronic disease prevention and control, and to achieve information sharing, resource mutual assistance, and policy linkage. The second is to promote the regional balanced layout of high-quality medical resources. The spillover effect of medical resources in central cities indicates that promoting the extension of medical resources to surrounding cities through regional medical centers, medical consortia, remote healthcare, and other means can effectively improve the overall health level. The government should encourage high-quality medical institutions to establish branch offices or carry out technology exports within urban areas, and strengthen the spatial radiation capability of medical resources. The third is to strengthen the coordination of health management and services under the background of population mobility. With the frequent cross city migration of population, it is necessary to improve the mutual recognition mechanism of health records of migrant population, promote the integration of medical settlement, health examination and disease management services in different places, and reduce the interruption of health services and the spread of health risks caused by migration. In summary, the spatial spillover characteristics of public health require policies to shift from “single city governance” to “regional collaborative governance,” forming a cross regional public health community through resource sharing, institutional co construction, and data interconnection, and achieving the overall promotion of the Healthy China strategy. In addition, under the background of integrated development of urban agglomerations and metropolitan areas, there is a clear regional synergy in public health governance. The health policies, environmental governance standards, and medical cooperation mechanisms introduced by higher-level governments have cross city transmission effects, resulting in a linkage change in public health levels between different cities.
2. Urban public cultural services are important public goods that improve residents’ quality of life, enhance social cohesion, and promote mental health. They have a significant positive impact on the level of public health. The following policy recommendations are proposed from the aspects of improving the public cultural supply system, promoting the integration and development of cultural health, and optimizing the mechanism of cultural benefits for the people: firstly, improving the urban public cultural service system and expanding the supply of high-quality cultural resources. The government should continue to increase investment in public cultural infrastructure, optimize the layout of cultural facilities such as public libraries, cultural centers, community cultural centers, and urban study rooms, and promote the extension of high-quality cultural resources to grassroots communities. Especially in densely populated areas and old communities, the construction of convenient cultural spaces should be increased to ensure that residents have easy access to cultural services, thereby promoting psychological relaxation and social participation, and improving overall health levels. The second is to promote the integrated development of “culture + health” and expand the function of cultural services for health. Encourage public cultural institutions to collaborate with health departments, explore new forms of services such as cultural and artistic healing, mental health lectures, and health themed exhibitions, and embed cultural activities into health promotion and disease prevention systems. For example, through activities such as music therapy, artistic creation, and book sharing, residents can reduce stress and improve their psychological state, achieving the integration goal of “cultural nourishment of body and mind, art promoting health.” Thirdly, we will increase efforts to benefit the people through culture and narrow the regional gap in cultural services. To address the issue of uneven distribution of public cultural resources between different regions, efforts should be made to promote cross regional flow of public cultural resources through fiscal transfer payments, targeted support, and digital platform construction. Utilizing digital cultural services such as online exhibitions, digital libraries, and cloud-based performances to enhance cultural accessibility for residents in underdeveloped areas and promote convergence of public health levels in a larger spatial scope. The fourth is to establish a linkage mechanism between cultural service performance and health outcomes. In the evaluation of urban public cultural policies, the “health impact assessment” indicator should be introduced to incorporate residents’ mental health, social participation, and life satisfaction into the cultural service performance evaluation system, promoting the transformation of public cultural policies towards a “culture health” collaborative orientation. Policy implications should be understood quantitatively rather than normatively. The elasticity estimates suggest that incremental increases in cultural service provision—such as improving the quality and accessibility of libraries, museums, and cultural centers—can produce tangible health benefits equivalent in scale to changes in core socio-economic variables like income or infrastructure. Hence, investments in cultural public goods can serve as complementary levers of public health promotion, particularly when designed to enhance social interaction and mental wellbeing. Future policy experiments could further validate these channels using quasi-experimental or longitudinal designs.

In short, enhancing public cultural services is not only a livelihood project that meets spiritual and cultural needs, but also an important support for promoting the Healthy China strategy of “physical and mental health, social harmony.” By promoting the construction of a cultural service system, promoting the integration of cultural health, and ensuring fair access to cultural resources for all, the policy goal of “promoting health through culture and happiness through health” can be achieved at a deeper level. The integration of culture and health at the local government level provides an important institutional pathway linking cultural service provision to health outcomes. In practice, many cities have begun to embed health elements into public cultural infrastructure, such as establishing community cultural centers that deliver health education, organizing wellness-oriented cultural events, or developing digital cultural platforms that disseminate health-related information. These initiatives not only enhance citizens’ access to both cultural and health resources but also promote healthy behaviors and lifestyles through cultural engagement. Consequently, local governments play a pivotal role in transforming cultural policy from symbolic enrichment into a practical driver of public health improvement.

## Data Availability

Publicly available datasets were analyzed in this study. This data can be found at: http://www.tjnjw.com/hangye/c/zhongguo-chengshi-tongjinianjian.html.
